# Problematic Use of Video Games, Social Media, and Alcohol: Exploring Reciprocal Relations with the Big Five Personality Traits in a Longitudinal Design

**DOI:** 10.3390/ejihpe15050077

**Published:** 2025-05-12

**Authors:** Lutz Wartberg, Steffen Zitzmann, Silke Diestelkamp, Katrin Potzel, Sophia Berber, Rudolf Kammerl

**Affiliations:** 1 Department of Psychology, Faculty of Human Sciences, MSH Medical School Hamburg, 20457 Hamburg, Germany; 2 Department of Education, Chair for Pedagogy with a Focus on Media Education, Friedrich-Alexander-University Erlangen-Nuremberg, 90478 Nuremberg, Germany

**Keywords:** gaming disorder, internet addiction, social media addiction, alcohol, adolescent, openness to experience, conscientiousness, extraversion, agreeableness, neuroticism

## Abstract

Background/Objectives: The problematic use of video games (PG), social media (PSMU), and alcohol (PAU) is widespread from adolescence onwards. According to theoretical models, personality traits are relevant for these problematic behavioral patterns; however, only very few longitudinal studies are available. The aim of this longitudinal study was to investigate for the first time whether Big Five personality dimensions (BFPD) are predictors for the development of PG, PSMU, or PAU, or conversely, whether these behavioral patterns are predictive of the BFPD. Methods: Surveys were conducted over three measurement time points (t1 to t3) using standardized instruments on PG, PSMU, PAU, and BFPD. A total of 492 young people (average age: 16.83 years, 44.1% female and 55.9% male) were investigated at t1, 475 persons (mean age: 17.93 years, 44.8% female, 55.2% male) at t2, and 443 cases (average age: 20.11 years, 45.1% female, 54.9% male) at t3. We calculated cross-lagged panel analyses over three measurement points (structural equation modeling). Results: Of the BFPD, lower Conscientiousness and lower Extraversion were predictors of PG, higher Negative Emotionality (Neuroticism) predicted PSMU, and lower Agreeableness was a predictor of PAU. Only PAU was a predictor of a Big Five dimension (lower Agreeableness). Conclusions: The findings were not consistent across the measurement points (t1 to t2 vs. t2 to t3) with one exception in an explorative analysis: problematic gaming was a predictor for both problematic social media use and problematic alcohol use in youth (t1 to t2 and t2 to t3). The influence of lower Conscientiousness was confirmed for PG and initial longitudinal results for PSMU and PAU were observed. These novel findings could be considered when developing or revising preventive measures.

## 1. Introduction

The use of video games and social media applications are among the most popular leisure activities for young people, both internationally and in Germany (where this study was conducted). The number of gamers worldwide continues to rise and, according to a current forecast, will reach a new high of over three billion persons (Statista, 2024c). In Germany, 91% of 16- to 29-year-olds play video games at least occasionally (Statista, 2024a). The number of people using social media was 5.04 billion worldwide in 2024 (Statista, 2024b). In 2024, 92% of 14- to 29-year-olds in Germany used social media applications at least once a week (Müller, 2024). With the inclusion of the (research) diagnosis of Internet Gaming Disorder in the appendix of the DSM-5, a disorder pattern relating to the use of digital media was included in an important classification system for the first time (American Psychiatric Association, 2013). The clear majority of players use video games as a leisure activity, but according to the available empirical findings, a minority appears to develop problematic patterns of use (e.g., Stevens et al., 2021). Even though it was not included as a diagnosis in the DSM-5 up to now, many empirical findings also indicate a potential for problematic use of social media (e.g., Mérelle et al., 2017). Worldwide, Stevens et al. (2021) estimated the prevalence of problematic gaming at 3.05% and Cheng et al. (2021) for problematic social media use at 5%. In the meantime, in the current version of the International Classification of Diseases (ICD-11), the WHO has also included a new category “Disorders due to addictive behaviors” (non-substance-related disorders) for the first time alongside the established category “Disorders due to substance” (substance-related disorders) (Reed et al., 2022). In the new category “Disorders due to addictive behaviors” (often also designated as addictive behaviors in the scientific literature), problematic use of video games in the form of the diagnosis “Gaming Disorder” can be classified (Reed et al., 2022). In addition to non-substance-related disorders, substance-related disorders are still widespread. For instance, in a representative sample of 12- to 17-year-olds in Germany, 5.0% were affected by problematic alcohol use (Wartberg et al., 2019), and during the COVID-19 pandemic, a prevalence estimate as high as 11.3% was observed in this age group (Wartberg et al., 2024).

An important model for the development of addictive behaviors (which also incorporate the problematic use of video games or social media) is the Interaction of Person–Affect–Cognition–Execution (I-PACE) model (Brand et al., 2016, 2019). The I-PACE model explicitly mentions personality traits for the development of addictive behaviors (Brand et al., 2019). According to Brand et al. (2016), for personality factors, the most consistent associations were reported for “… high impulsivity, low self-esteem, low conscientiousness, high shyness, high neuroticism, a tendency to procrastinate, and low self-directedness” (p. 255). Furthermore, following Brand et al. (2016), it is likely that different types of addictive behaviors “… are linked to specific personality traits” (p. 256). Consequently, personality dimensions should be considered in studies on the etiology of different addictive behaviors (e.g., Brand et al., 2019). The best-known and most frequently used approach to measure personality dimensions worldwide is the five-factor model (often abbreviated as the Big Five) (McCrae & Costa, 1997). The Big Five have been empirically confirmed across different cultures with their personality dimensions Openness to experience, Conscientiousness, Extraversion, Agreeableness, and Neuroticism, commonly abbreviated by the acronym OCEAN. These five personality dimensions can be briefly explained with the following: fanciful or active imagination (Openness to experience), thoroughness vs. convenience (Conscientiousness), sociable vs. reserved (Extraversion), a tendency to criticize others vs. trust others (Agreeableness), and nervous, insecure vs. relaxed (Neuroticism) (Groß & Kohlmann, 2018). There are various established instruments that are used internationally to assess the Big Five, e.g., the NEO-Five-Factor Inventory (NEO-FFI, Costa & McCrae, 1989) or the Big Five Inventory 2 (BFI-2, Soto & John, 2017).

There is some empirical evidence on the relationship between the Big Five personality dimensions and the problematic use of video games (abbreviated in the following with problematic gaming), but most of it comes from cross-sectional studies (Şalvarlı & Griffiths, 2021). According to the systematic review by Şalvarlı and Griffiths (2021), there is a lack of longitudinal studies, although the very first longitudinal findings have been published in the meantime. For young people (who also made up the sample in the present study), associations between lower levels of Conscientiousness and problematic gaming were most commonly reported in the literature as the results of cross-sectional studies (López-Fernández et al., 2021a, 2021b; Rodríguez-Ruiz et al., 2023; Sánchez-Llorens et al., 2023; Wang et al., 2015). A higher Neuroticism was related to problematic gaming in the cross-sectional surveys of López-Fernández et al. (2021b), Rodríguez-Ruiz et al. (2023), Vollmer et al. (2014), and Wartberg et al. (2023b). Cross-sectional relations between lower Agreeableness and problematic gaming were observed by López-Fernández et al. (2021a), Sánchez-Llorens et al. (2023), Rodríguez-Ruiz et al. (2023), and Vollmer et al. (2014). A lower Openness to experience was cross-sectionally associated with problematic gaming according to Rodríguez-Ruiz et al. (2023), Wang et al. (2015), and Wartberg et al. (2023b). Relationships between lower Extraversion and problematic gaming were found in two cross-sectional studies (Rodríguez-Ruiz et al., 2023; Vollmer et al., 2014). The initial results from the first longitudinal surveys showed lower Conscientiousness and higher Extraversion (Ok, 2021), lower Conscientiousness and lower Agreeableness (Marrero et al., 2021), and lower Conscientiousness (Jhang, 2024) as predictors for problematic gaming.

Regarding associations with the Big Five, there are fewer findings for the problematic use of social media or alcohol in adolescence. More pronounced Neuroticism and Extraversion in problematic social media use were found by Wang et al. (2015) and Wartberg et al. (2023b) respectively. Relations between higher Neuroticism and problematic alcohol use were also reported (Sánchez-Llorens et al., 2023; Wartberg et al., 2023b). Thus far, relationships between problematic alcohol use and lower Conscientiousness and Agreeableness (Ibáñez et al., 2015) as well as lower Openness (Wartberg et al., 2023b) have only been observed in one study in each case.

In summary, according to the I-PACE model, personality dimensions (including Conscientiousness and Neuroticism, see, e.g., Brand et al., 2016) should be considered when investigating the etiology of addictive behaviors. Furthermore, Şalvarlı and Griffiths (2021) emphasized that the results from longitudinal studies in which the Big Five are examined as possible predictors of problematic gaming are particularly lacking (such a lack of longitudinal results also applies to the problematic use of social media and alcohol or is even more pronounced). In the few published longitudinal surveys (Jhang, 2024; Marrero et al., 2021; Ok, 2021), consistently lower Conscientiousness was a predictor for problematic gaming; concerning the other four Big Five dimensions, however, the findings were heterogeneous. With regard to the Big Five, there are no longitudinal findings of this kind on the problematic use of social media and alcohol among young people. As far as we are aware, there are no studies to date that have not only examined whether the Big Five are predictors for problematic use of video games, social media, or alcohol but also whether these behavioral patterns could predict the Big Five. In an exploratory analysis, the longitudinal potential of the data was also used to examine how the problematic use of video games, social media, and alcohol influence each other over time. In this study, we investigated the following research questions:

(I) Are one or more dimensions of the Big Five (Open-Mindedness, Conscientiousness, Extraversion, Agreeableness, and Negative Emotionality) predictors for problematic gaming?
(II) Is problematic gaming a predictor for one or more dimensions of the Big Five?
(III) Are one or more dimensions of the Big Five predictors for problematic social media use?
(IV) Is problematic social media use a predictor for one or more dimensions of the Big Five?
(V) Are one or more dimensions of the Big Five predictors for problematic alcohol use?
(VI) Is problematic alcohol use a predictor for one or more dimensions of the Big Five?
(VII) How do problematic gaming, problematic social media use, and problematic alcohol use mutually influence each other over time?

## 2. Materials and Methods

### 2.1. Procedure

Data were collected in the VEIF project, a longitudinal study assessing problematic digital media use among children and adolescents in Germany. The analyses used data from three measurement time points collected between the years 2020 and 2023 (t1: January to March 2020; t2: January to April 2021; and t3: May to June 2023). The Ethics Committee of the German Educational Research Association (GERA, German designation: Deutsche Gesellschaft für Erziehungswissenschaft, DGfE) provided ethics approval (the approval number for t1 and t2 was 01/2018/DGfE and the approval code for t3 was 01/2021/DGfE). Data collection was implemented face-to-face with the interviewers visiting the participating families in their homes (t1: 82 interviewers, t2: 77 persons, and t3: 65 interviewers). The VEIF project was drafted to recruit and study a sample with an increased risk of problematic use of digital media in comparison to the general population. Therefore, the recruitment procedure was designed to yield a sample in which adolescents with an increased risk of problematic use of digital media were oversampled.

### 2.2. Measures

Problematic gaming was assessed through self-ratings of youths’ gaming behavior in the past 12 months by applying the Internet Gaming Disorder Scale (IGDS; Lemmens et al., 2015). The youths were asked to answer nine questions with a yes/no response format (0 = ‘no’, 1 = ‘yes’). A total score ranging from 0 to 9 was calculated from the answers to the items. A higher score reflects a higher level of problematic gaming. The IGDS was recommended as a measurement instrument in systematic reviews based on its good psychometric properties (King et al., 2020; Yoon et al., 2021). In the present study, the IGDS showed good reliability across the measurement time points with coefficients (Cronbach’s alpha) between 0.83 (t1), 0.83 (t2), and 0.86 (t3).

The youth provided information on their problematic social media use (past 12 months) by answering the Social Media Disorder Scale (SMDS; van den Eijnden et al., 2016). The scale comprises nine items with a binary response format (0 = ‘no’, 1 = ‘yes’), which yields a total sum score ranging between 0 and 9. A higher sum score reflects a higher level of problematic social media use. For the SMDS, good psychometric properties were observed in a comprehensive cross-national validation study (Boer et al., 2022). In the present survey, Cronbach’s alpha showed good internal consistency for the scale across the measurement time points [0.80 (t1), 0. 82 (t2), and 0.82 (t3)].

The Alcohol Use Disorders Identification Test-Consumption (AUDIT-C; Bush et al., 1998) was used to assess problematic alcohol use. The AUDIT-C is a self-rating instrument consisting of three questions on problematic alcohol use within the past 12 months. It provides an alternating five-point response format. The AUDIT-C is recommended as a screening tool for adolescents (Rumpf et al., 2013) and in the German Guideline for Screening, Diagnosis, and Treatment of Alcohol-Related Disorders (Kiefer et al., 2021). AUDIT-C starts with assessing how often respondents had a drink containing alcohol in the past 12 months with a response format ranging from 0 = ‘never’, 1 = ‘monthly or less’, 2 = ‘2 to 4 times a month’, 3 = ‘2 to 3 times a week’, to 4 = ‘4 or more times a week’. The second item of the AUDIT-C relates to the quantity of consumed alcohol and asks how many drinks containing alcohol respondents consume on a typical day when they are drinking. The five-level response format ranges from 0 = ‘1 to 2’, 1 = ‘3 to 4’, 2 = ‘5 to 6’, 3 = ‘7 to 9’, to 4 = ‘10 or more’. The third AUDIT-C item assesses the frequency of binge drinking. Respondents provide information on how often they consume six or more drinks on one occasion. The respective response format has the following answering options: 0 = ‘never’, 1 = ‘less than monthly’, 2 = ‘monthly’, 3 = ‘weekly’, 4 = ‘daily or almost daily’. The resulting sum score ranges from 0 to 12 with a higher sum reflecting a higher level of problematic alcohol use. The reliability coefficients of the AUDIT-C in the sample were as follows: 0.63 (t1), 0.59 (t2), and 0.69 (t3).

The five domains of personality according to the Big Five model of personality (Open-Mindedness, Conscientiousness, Extraversion, Agreeableness, and Negative Emotionality were measured with the German version (Danner et al., 2019) of the established Big Five Inventory-2 (BFI-2, Soto & John, 2017). The 60 items of the BFI-2 have a five-level response format (1 = ‘Disagree strongly’, 2 = ‘Rather disagree’, 3 = ‘Partly, partly’, 4 = ‘Rather agree’, and 5 = ‘Agree strongly’) (Danner et al., 2019). Each personality domain is assessed with 12 items. A mean value ranging between 1 and 5 can be calculated for each personality domain. A higher mean value reflects a higher expression of this personality trait. A recently published validation study showed consistently a high psychometric quality for BFI-2 versions in different languages (Rammstedt et al., 2024). In the present survey, Cronbach’s alpha for the BFI-2 across the measurement time points were as follows: 0.81 (t1), 0.76 (t2), and 0.81 (t3) for Open-Mindedness; 0.88 (t1), 0.82 (t2), and 0.87 (t3) for Conscientiousness; 0.79 (t1), 0.76 (t2), and 0.80 (t3) for Extraversion; 0.84 (t1), 0.79 (t2), and 0.81 (t3) for Agreeableness; and 0.81 (t1), 0.78 (t2), and 0.82 (t3) for Negative Emotionality.

### 2.3. Sample Description

At the first measurement time point (t1), a total of 492 adolescents had provided data for the longitudinal VEIF study with 44.1% identifying as female and 55.9% as male. At t2, the sample comprised 475 participants (44.8% female, 55.2% male), and at the third measurement time point (t3), a total of 443 youth had participated in the assessment (45.1% female, 54.9% male). In the first assessment (t1), the participants reported a mean age of 16.83 years (SD = 0.97). In the following two assessments, the participants reported average ages of 17.93 years (SD = 1.01) (t2) and 20.11 years (SD = 0.91) (t3), respectively. At t1, the majority of the participants attended school (76.4%) while 23.0% reported to have finished school. A minority of 0.6% were not currently attending school without having completed it. At the third measurement time point (t3), this situation had changed and 82.6% reported to have graduated from school while only 16.5% still attended school (0.9% did not currently go to school without having a graduation). Among those who reported to have graduated from school at t3, the majority (56.7%) had achieved a medium education level, followed by 34.2% who achieved a high education level and 8.8% who had achieved a low education level. A very small percentage of 0.3% of the study participants had left school without graduation at t3.

### 2.4. Statistical Analyses

The statistical software IBM SPSS Statistics (Version 27) was used to compute means, standard deviations, frequencies, reliability coefficients (Cronbach’s alpha), and correlations. For our cross-lagged panel analyses, we computed structural equation models (SEMs) using Mplus version 8.10 (Muthén & Muthén, 2017). In the first SEM, we used data on problematic gaming, Open-Mindedness, Conscientiousness, Extraversion, Agreeableness, and Negative Emotionality from the second (t2) and third (t3) measurement time points as lagged variables. The same variables (problematic gaming, Open-Mindedness, Conscientiousness, Extraversion, Agreeableness, and Negative Emotionality) were used as explanatory variables (all examined one measurement time point beforehand, for t2 at t1 and for t3 at t2). In the second SEM, problematic social media use was analyzed instead of problematic gaming while the same other lagged and explanatory variables were used in the model. The same approach was used for the third SEM in which problematic alcohol use was investigated with the same lagged and explanatory variables as in models 1 and 2. In an additional explorative cross-lagged panel analysis, we calculated a further SEM to examine the relationships between the three problematic behavioral patterns (problematic gaming, problematic social media use, and problematic alcohol use) over time. We determined goodness-of-fit indices for each model (the root-mean-square error of approximation or RMSEA, the standardized root-mean-square residual or SRMR, the Comparative Fit Index or CFI, and the Tucker–Lewis index or TLI).

## 3. Results

### 3.1. Results of the Correlation Analyses

The means and standard deviations of the variables used are presented in Table 1. The empirical findings of the correlational analyses are shown in Table 2. We observed numerous statistically significant correlations between problematic gaming and all the Big Five personality domains (Open-Mindedness, Conscientiousness, Extraversion, Agreeableness, and Negative Emotionality) at and between the different measurement time points (see Table 2). For example, problematic gaming at t3 was associated with all the Big Five domains at all the measuring points. For problematic social media use, there were also many significant relations with the Big Five domains. However, the findings for Open-Mindedness, Extraversion, and Agreeableness were heterogeneous (see also Table 2). Furthermore, we found relationships between problematic alcohol use and Conscientiousness, Agreeableness, and Negative Emotionality (see Table 2). There were mixed findings regarding the associations with Open-Mindedness. But, we observed no significant relationship between problematic alcohol use and Extraversion (see also Table 2).

### 3.2. Results of the Cross-Lagged Analyses for Problematic Gaming and the Big Five Domains

In the cross-lagged analyses, problematic gaming at t1 and lower Conscientiousness at t1 predicted problematic gaming at t2 (see Table 3). Problematic gaming at t2 and lower Extraversion at t2 were predictors for problematic gaming at t3. The other personality domains (Open-Mindedness, Agreeableness, and Negative Emotionality) did not predict problematic gaming. Conversely, problematic gaming was no significant predictor of any Big Five personality domain (see also Table 3).

### 3.3. Results of the Cross-Lagged Analyses for Problematic Social Media Use and the Big Five Domains

We found problematic social media use at t1 and stronger Negative Emotionality at t1 to be predictive of problematic social media use at t2 (see Table 4). Problematic social media use at t2 was the only significant predictor of problematic social media use at t3. Problematic social media was also no significant predictor of any Big Five personality domain (see also Table 4).

### 3.4. Results of the Cross-Lagged Analyses for Problematic Alcohol Use and the Big Five Domains

We observed problematic alcohol use (t1) to be predictive of problematic alcohol use (t2) and lower Agreeableness (t2, see Table 5). Problematic alcohol use at t2 and lower Agreeableness at t2 predicted problematic alcohol use at t3 (see also Table 5).

### 3.5. Results of the Cross-Lagged Analyses for the Three Problematic Behavioral Patterns

Problematic gaming at t1 and problematic social media use at t1 predicted problematic gaming at t2 (see Table 6), while problematic gaming at t2 was the only significant predictor of problematic gaming at t3. We found both problematic gaming at t1 and problematic social media use at t1 to be predictive of problematic social media use at t2 (see also Table 6). Problematic gaming at t2 and problematic social media use at t2 also predicted problematic social media use at t3. Furthermore, we observed problematic gaming at t1 and problematic alcohol use at t1 to be predictive of problematic alcohol use at t2 (see Table 6). Problematic gaming (t2) and problematic alcohol use (t2) were also predictors for problematic alcohol use (t3).

## 4. Discussion

In the present study, reciprocal longitudinal relations between the Big Five dimensions and problematic use of video games, social media, or alcohol were investigated for the first time (using a cross-lagged panel design with three measurement points). Prior to this, there were only a few findings from longitudinal studies in which the Big Five dimensions had been examined as predictors of problematic gaming (Jhang, 2024; Marrero et al., 2021; Ok, 2021). Analogous to the studies by Ok (2021), Marrero et al. (2021), and Jhang (2024), lower Conscientiousness at t1 was shown to be a predictor for problematic gaming at t2. In general, Conscientiousness seems to have a relatively high relevance for problematic use of video games. The meta-analysis by Chew (2022) showed the strongest relations between Conscientiousness and problematic gaming, taking all the dimensions of the Big Five into account (however, the meta-analysis was based almost exclusively on cross-sectional studies and did not differentiate between minors and adults).

In contrast, between the next two measurement times, concerning the Big Five at t2, only lower Extraversion was a predictor for problematic gaming at t3 in the present study. This difference could be due to the age of the sample. The sample in our study was on average 20.11 years old at t3 and thus significantly older, especially in comparison to the adolescents from the published longitudinal investigations by Marrero et al. (2021) and Jhang (2024). It is conceivable that different Big Five dimensions could be relevant to the development of problematic gaming at different points in young people’s lives. It was previously unclear whether problematic gaming could predict one or more dimensions of the Big Five, but there were no empirical indications of this in this survey.

Longitudinal findings regarding the Big Five dimensions as predictors of problematic social media use have so far been lacking. Accordingly, only the results from cross-sectional studies can be used to classify the results of the present survey. These cross-sectional findings showed associations between problematic social media use and more pronounced Extraversion (Wang et al., 2015; Wartberg et al., 2023b) and Neuroticism (Wang et al., 2015) or Negative Emotionality (Wartberg et al., 2023b). A higher Negative Emotionality at t1 (in the BFI-2, the designation Negative Emotionality is used instead of Neuroticism) was also a predictor for problematic social media use at t2 in the present longitudinal study. A higher Negative Emotionality could encourage problematic social media use by using it as a strategy to cope with stress (e.g., to relieve tension) or to regulate (negative) emotions. However, it should also be noted for problematic social media use that although Negative Emotionality is a predictor for this behavioral pattern from t1 to t2, this finding is not evident between t2 and t3. In addition to the aforementioned idea of different predictors at different points in young people’s lives, it could also be assumed that the intervals between the measurement time points were not uniform (about 1 year between t1 and t2 versus more than two years between t2 and t3), which could possibly have favored differences in the results. There was also no empirical evidence for problematic social media use as a predictor for one or more Big Five dimensions in the present examination.

For problematic alcohol use in young people, there was also a lack of preliminary longitudinal findings regarding the Big Five as predictors. In the cross-sectional study of Ibáñez et al. (2015), lower Agreeableness was associated with problematic alcohol use. The present survey showed a very interesting result, namely problematic alcohol use at t1 as a predictor for lower Agreeableness at t2 and lower Agreeableness at t2 as a predictor for problematic alcohol use at t3 (as a kind of reciprocal effect, even if not between the same measurement time points). One possible explanation could be that the psychotropic effects of alcohol encourage certain actions (e.g., aggressive behavior), but that the consumption of alcohol may also be used deliberately, for example, to facilitate social interactions (especially in adolescence and among young adults, alcohol is often consumed in social situations or in a group context). Like the results for problematic gaming and problematic social media use, these very interesting empirical findings for problematic alcohol use naturally require further examination in future longitudinal studies.

In an additional exploratory analysis, we explored how problematic gaming, problematic social media use, and problematic alcohol use mutually influence each other over time. Such associations have been studied extremely rarely in youth to date, and the first cross-sectional results showed positive correlations between these problematic behavioral patterns (Wartberg & Kammerl, 2020). In adults, there are also initial cross-sectional findings that show relationships between problematic alcohol use and problematic social media use (Lyvers et al., 2019) as well as between problematic alcohol use and problematic gaming (Na et al., 2017), although the results are not uniform (Erevik et al., 2019). The novel longitudinal findings in the present study are particularly interesting, according to which problematic gaming was a predictor for both problematic social media use and problematic alcohol use in youth (between t1 and t2 as well as between t2 and t3). According to the Problem Behavior Theory of Jessor (1991), problematic behavioral patterns often do not occur in isolation, but together, and these results also support this for problematic gaming, problematic social media use, and problematic alcohol use. Here too, of course, further investigations are needed to re-examine the findings.

The present study has various limitations. This longitudinal survey (in the context of the VEIF project) does not examine a representative sample, but a sample that showed an increased risk of excessive use of digital media at least before the first data collection (in 2016). The extent to which the reported results are transferable to the general population cannot be conclusively assessed. With regard to the fit indices of the computed structural equation models (see Note below Table 3, Table 4, Table 5 and Table 6 each), the findings are somewhat heterogeneous. For example, according to Sathyanarayana and Mohanasundaram (2024), the model presented in Table 5 shows good values for CFI and SRMR (indicating a good fit of this model), the RMSEA value is acceptable, and the value for TLI is quite lower. However, in this common design for measuring cross-lagged effects, it is difficult to justify a change in the selection of variables (e.g., concerning the Big Five). Another limitation is the self-report of the participants. Alternatively, or additionally, it would be conceivable to obtain an external assessment [e.g., see Wiechers and Kandler (2025) for empirical results for the BFI-2]. Furthermore, we were not able to formulate a prediction model from the data; in particular, the heterogeneous findings concerning the Big Five domains as predictors for the problematic use of video games, social media, or alcohol made this difficult. Deriving such prediction models from future longitudinal studies would be an important step towards a better etiologic understanding of these behavioral patterns. According to the available empirical findings, in addition to the personality dimensions, other characteristics of young people are also relevant for problematic use of video games, social media, or alcohol, for example, psychopathological burden (e.g., Wartberg & Kammerl, 2020) or family aspects [e.g., Vossen et al. (2024) for problematic social media use and Wartberg et al. (2023a) for problematic gaming and alcohol use]. All these aspects should be further investigated in combination with future studies (ideally also in longitudinal designs). In addition to the five-factor model, there are also alternative models of central personality traits, such as the HEXACO model (Ashton & Lee, 2007) which consists of six factors.

## 5. Conclusions

Despite the aforementioned limitations, the present study offers novel and interesting findings that may contribute to a better understanding of the etiology of problematic use of video games, social media, or alcohol in young people, particularly through the longitudinal design. Especially, the observed interactions between the three problematic behavioral patterns are extremely relevant, and should these findings be confirmed, these results must be taken into account in the development or revision of preventive approaches for minors or young adults.

## Figures and Tables

**Table 1:** Means and standard deviations for the problematic behavioral patterns and the Big Five personality domains.

Variable	t1	t2	t3
	M (SD)	M (SD)	M (SD)
(1) Internet gaming disorder	1.48 (2.18)	1.20 (1.98)	0.94 (1.89)
(2) Problematic social media use	1.19 (1.88)	0.85 (1.69)	0.63 (1.48)
(3) Problematic alcohol use	2.10 (1.99)	2.00 (1.80)	2.56 (2.10)
(4) Open-Mindedness (BFI-2)	3.12 (0.63)	3.12 (0.58)	3.15 (0.62)
(5) Conscientiousness (BFI-2)	3.53 (0.67)	3.39 (0.61)	3.47 (0.67)
(6) Extraversion (BFI-2)	3.44 (0.57)	3.32 (0.54)	3.29 (0.59)
(7) Agreeableness (BFI-2)	3.77 (0.59)	3.54 (0.56)	3.61 (0.56)
(8) Negative Emotionality (BFI-2)	2.38 (0.57)	2.56 (0.55)	2.48 (0.60)

Note. t1 = time point 1; t2 = time point 2; t3 = time point 3. M = mean; SD = standard deviation. BFI-2 = Big Five Inventory-2.

**Table 2:** Correlation matrix for the problematic behavioral patterns and the Big Five personality domains over the three measurement points.

Variable	1	2	3	4	5	6	7	8	9	10	11	12	13	14	15	16	17	18	19	20	21	22	23	24
(1) Internet Gaming Disorder (t1)	—																							
(2) Internet Gaming Disorder (t2)	0.56 ***	—																						
(3) Internet Gaming Disorder (t3)	0.49 ***	0.49 ***	—																					
(4) Problematic Social Media Use (t1)	0.44 ***	0.34 ***	0.23 ***	—																				
(5) Problematic Social Media Use (t2)	0.37 ***	0.63 ***	0.32 ***	0.49 ***	—																			
(6) Problematic Social Media Use (t3)	0.35 ***	0.39 ***	0.55 ***	0.42 ***	0.46 ***	—																		
(7) Problematic Alcohol Use (t1)	0.13 **	0.06	0.10	0.07	0.02	0.05	—																	
(8) Problematic Alcohol Use (t2)	0.17 ***	0.24 ***	0.15 **	0.08	0.11 *	0.06	0.47 ***	—																
(9) Problematic Alcohol Use (t3)	0.17 ***	0.17 ***	0.21 ***	0.08	0.02	0.11 *	0.36 ***	0.38 ***	—															
(10) Open-Mindedness (t1)	−0.27 ***	−0.17 ***	−0.18 ***	−0.09	−0.05	−0.05	−0.14 **	−0.16 ***	−0.13 **	—														
(11) Open-Mindedness (t2)	−0.16 ***	−0.24 ***	−0.20 ***	−0.09	−0.14 **	−0.06	−0.07	−0.09	−0.14 **	0.59 ***	—													
(12) Open-Mindedness (t3)	−0.14**	−0.21 ***	−0.29 ***	−0.07	−0.13 **	−0.16 ***	−0.07	−0.02	−0.22 ***	0.39 ***	0.51 ***	—												
(13) Conscientiousness (t1)	−0.34 ***	−0.31 ***	−0.31 ***	−0.19 ***	−0.19 ***	−0.22 ***	−0.17 ***	−0.19 ***	−0.18 ***	0.48 ***	0.30 ***	0.31 ***	—											
(14) Conscientiousness (t2)	−0.16 ***	−0.30 ***	−0.22 ***	−0.08	−0.16 ***	−0.10 *	−0.15 **	−0.17 ***	−0.18 ***	0.34 ***	0.52 ***	0.35 ***	0.49 ***	—										
(15) Conscientiousness (t3)	−0.17 ***	−0.19 ***	−0.38 ***	−0.12 *	−0.11 *	−0.25 ***	−0.16 ***	−0.13 **	−0.29 ***	0.22 ***	0.27 ***	0.62 ***	0.40 ***	0.56 ***	—									
(16) Extraversion (t1)	−0.16 ***	−0.16 ***	−0.20 ***	−0.06	−0.14 **	−0.19 ***	−0.00	−0.06	−0.07	0.38 ***	0.26 ***	0.21 ***	0.39 ***	0.22 ***	0.18 ***	—								
(17) Extraversion (t2)	−0.05	−0.18 ***	−0.23 ***	−0.07	−0.16 ***	−0.12 *	0.02	−0.00	−0.03	0.27 ***	0.52 ***	0.31 ***	0.20 ***	0.37 ***	023 ***	0.41 ***	—							
(18) Extraversion (t3)	−0.06	−0.15 **	−0.27 ***	0.01	−0.12 *	−0.18 ***	−0.07	−0.02	−0.06	0.28 ***	0.38 ***	0.59 ***	0.22 ***	0.30 ***	0.52 ***	0.39 ***	0.50 ***	—						
(19) Agreeableness (t1)	−0.31 ***	−0.27 ***	−0.24 ***	−0.19 ***	−0.17 ***	−0.18 ***	−0.15 ***	−0.14 **	−0.21 ***	0.21 ***	0.18 ***	0.19 ***	0.65 ***	0.28 ***	0.23 ***	0.24 ***	0.09	0.13 *	—					
(20) Agreeableness (t2)	−0.16 ***	−0.18 ***	−0.13 **	−0.12 **	−0.10 *	−0.01	−0.16 ***	−0.16 ***	−0.25 ***	0.31 ***	0.40 ***	0.18 ***	0.24 ***	0.60 ***	0.33 ***	0.16 ***	0.31 ***	0.22 ***	0.34 ***	—				
(21) Agreeableness (t3)	−0.12 *	−0.06	−0.26 ***	−0.05	0.01	−0.14 **	−0.25 ***	−0.13 **	−0.34 ***	0.19 ***	0.22 ***	0.41 ***	0.21 ***	0.42 ***	0.64 ***	0.12 *	0.15 **	0.40 ***	0.29 ***	0.51 ***	—			
(22) Negative Emotionality (t1)	0.30 ***	0.25 ***	0.21 ***	0.28 ***	0.30 ***	0.33 ***	0.09 *	0.12 **	0.11 **	−0.15 ***	−0.08	−0.15 **	−0.56 ***	−0.28 ***	−0.26 ***	−0.45 ***	−0.17 ***	−0.20 ***	−0.60 ***	−0.22 ***	−0.22 ***	—		
(23) Negative Emotionality (t2)	0.11 *	0.21 ***	0.12 *	0.15 **	0.22 ***	0.13 **	0.10 *	0.13 **	0.15 **	−0.15 **	−0.30 ***	−0.13 **	−0.23 ***	−0.56 ***	−0.33 ***	−0.17 ***	−0.38 ***	−0.21 ***	−0.20 ***	−0.61 ***	−0.36 ***	0.42 ***	—	
(24) Negative Emotionality (t3)	0.10	0.10 *	0.25 ***	0.17 ***	0.15 **	0.33 ***	0.17 ***	0.10 *	0.20 ***	−0.12 *	−0.13 **	−0.39 ***	−0.28 ***	−0.36 ***	−0.61 ***	−0.26 ***	−0.24 ***	−0.49 ***	−0.27 ***	−0.35 ***	−0.60 ***	0.49 ***	0.52 ***	—

Note. t1 = time point 1; t2 = time point 2; t3 = time point 3. *** *p* < 0.001; ** *p* < 0.01; * *p* < 0.05.

**Table 3:** Results of the structural equation model for problematic gaming.

Variable	Internet Gaming Disorder (t2) Beta Coefficient (SE)	Open-Mindedness (t2) Beta Coefficient (SE)	Conscientiousness (t2) Beta Coefficient (SE)	Extraversion (t2) Beta Coefficient (SE)	Agreeableness (t2) Beta Coefficient (SE)	Negative Emotionality (t2) Beta Coefficient (SE)	Internet Gaming Disorder (t3) Beta Coefficient (SE)	Open-Mindedness (t3) Beta Coefficient (SE)	Conscientiousness (t3) Beta Coefficient (SE)	Extraversion (t3) Beta Coefficient (SE)	Agreeableness (t3) Beta Coefficient (SE)	Negative Emotionality (t3) Beta Coefficient (SE)
Internet Gaming Disorder (t1)	0.51 *** (0.04)	−0.00 (0.04)	0.02 (0.04)	0.03 (0.05)	0.01 (0.05)	−0.03 (0.05)	—	—	—	—	—	—
Open-Mindedness (t1)	0.06 (0.05)	0.57 *** (0.04)	0.14 ** (0.05)	0.14 ** (0.05)	0.32 *** (0.05)	−0.15 ** (0.05)	—	—	—	—	—	—
Conscientiousness (t1)	−0.13 * (0.06)	−0.04 (0.06)	0.44 *** (0.06)	0.02 (0.07)	−0.20 ** (0.07)	0.04 (0.07)	—	—	—	—	—	—
Extraversion (t1)	−0.06 (0.05)	0.08 (0.04)	−0.00 (0.05)	0.36 *** (0.05)	0.01 (0.05)	0.08 (0.05)	—	—	—	—	—	—
Agreeableness (t1)	−0.02 (0.06)	0.12 * (0.05)	−0.07 (0.06)	−0.03 (0.06)	0.36 *** (0.06)	0.09 (0.06)	—	—	—	—	—	—
Negative Emotionality (t1)	−0.01 (0.05)	0.09 (0.05)	−0.05 (0.06)	−0.01 (0.06)	−0.07 (0.06)	0.51 *** (0.06)	—	—	—	—	—	—
Internet Gaming Disorder (t2)	—	—	—	—	—	—	0.46 *** (0.04)	−0.07 (0.04)	−0.01 (0.04)	−0.02 (0.04)	0.08 (0.04)	−0.03 (0.04)
Open-Mindedness (t2)	—	—	—	—	—	—	0.01 (0.06)	0.42 *** (0.05)	−0.05 (0.05)	0.12 * (0.05)	−0.00 (0.05)	0.10 (0.06)
Conscientiousness (t2)	—	—	—	—	—	—	−0.07 (0.06)	0.18 ** (0.06)	0.57 *** (0.05)	0.09 (0.06)	0.21 *** (0.06)	−0.12 * (0.06)
Extraversion (t2)	—	—	—	—	—	—	−0.15 ** (0.05)	0.06 (0.05)	0.03 (0.05)	0.41 *** (0.05)	−0.04 (0.05)	−0.07 (0.05)
Agreeableness (t2)	—	—	—	—	—	—	−0.01 (0.06)	−0.06 (0.06)	−0.01 (0.06)	0.03 (0.06)	0.41 *** (0.05)	−0.02 (0.06)
Negative Emotionality (t3)	—	—	—	—	—	—	−0.08 (0.06)	0.09 (0.06)	−0.01 (0.06)	0.05 (0.06)	−0.03 (0.06)	0.45 *** (0.05)
*R* ^2^	0.33	0.36	0.26	0.19	0.19	0.19	0.26	0.29	0.31	0.27	0.30	0.28

Note. Model fit: RMSEA = 0.09; SRMR = 0.05; CFI = 0.96; TLI = 0.83. t1 = time point 1; t2 = time point 2; t3 = time point 3. SE = standard error. *** *p* < 0.001; ** *p* < 0.01; * *p* < 0.05.

**Table 4:** Results of the structural equation model for problematic social media use.

Variable	Problematic Social Media Use (t2) Beta Coefficient (SE)	Open-Mindedness (t2) Beta Coefficient (SE)	Conscientiousness (t2) Beta Coefficient (SE)	Extraversion (t2) Beta Coefficient (SE)	Agreeableness (t2) Beta Coefficient (SE)	Negative Emotionality (t2) Beta Coefficient (SE)	Problematic Social Media Use (t3) Beta Coefficient (SE)	Open-Mindedness (t3) Beta Coefficient (SE)	Conscientiousness (t3) Beta Coefficient (SE)	Extraversion (t3) Beta Coefficient (SE)	Agreeableness (t3) Beta Coefficient (SE)	Negative Emotionality (t3) Beta Coefficient (SE)
Problematic Social Media Use (t1)	0.44 *** (0.04)	−0.04 (0.04)	0.01 (0.04)	−0.04 (0.04)	−0.05 (0.04)	0.03 (0.04)	—	—	—	—	—	—
Open-Mindedness (t1)	0.06 (0.05)	0.56 *** (0.04)	0.14 ** (0.05)	0.14 ** (0.05)	0.32 *** (0.05)	−0.14 ** (0.05)	—	—	—	—	—	—
Conscientiousness (t1)	−0.06 (0.06)	−0.04 (0.06)	0.44 *** (0.06)	0.02 (0.07)	−0.20 ** (0.07)	0.04 (0.07)	—	—	—	—	—	—
Extraversion (t1)	−0.05 (0.05)	0.09 (0.04)	−0.00 (0.05)	0.36 *** (0.05)	0.01 (0.05)	0.07 (0.05)	—	—	—	—	—	—
Agreeableness (t1)	0.05 (0.06)	0.12 * (0.05)	−0.07 (0.06)	−0.04 (0.06)	0.36 *** (0.06)	0.09 (0.06)	—	—	—	—	—	—
Negative Emotionality (t1)	0.16 ** (0.06)	0.10 (0.05)	−0.05 (0.06)	−0.01 (0.06)	−0.06 (0.06)	0.50 *** (0.06)	—	—	—	—	—	—
Problematic Social Media Use (t2)	—	—	—	—	—	—	0.45 *** (0.04)	−0.05 (0.04)	−0.03 (0.04)	−0.02 (0.04)	0.07 (0.04)	0.04 (0.04)
Open-Mindedness (t2)	—	—	—	—	—	—	0.05 (0.06)	0.43 *** (0.05)	−0.05 (0.05)	0.12 * (0.05)	−0.01 (0.05)	0.11 * (0.05)
Conscientiousness (t2)	—	—	—	—	—	—	−0.08 (0.06)	0.19 ** (0.06)	0.57 *** (0.05)	0.09 (0.06)	0.19 *** (0.06)	−0.12 * (0.06)
Extraversion (t2)	—	—	—	—	—	—	−0.04 (0.05)	0.06 (0.05)	0.02 (0.05)	0.41 *** (0.05)	−0.04 (0.05)	−0.07 (0.05)
Agreeableness (t2)	—	—	—	—	—	—	0.11 (0.06)	−0.06 (0.06)	−0.00 (0.06)	0.03 (0.06)	0.40 *** (0.05)	−0.03 (0.06)
Negative Emotionality (t3)	—	—	—	—	—	—	0.05 (0.06)	0.10 (0.06)	−0.01 (0.06)	0.05 (0.06)	−0.04 (0.06)	0.43 *** (0.06)
*R* ^2^	0.27	0.36	0.26	0.19	0.20	0.20	0.22	0.29	0.32	0.27	0.29	0.28

Note. Model fit: RMSEA = 0.09, SRMR = 0.05, CFI = 0.96, TLI = 0.84. t1 = time point 1, t2 = time point 2, t3 = time point 3. SE = standard error. *** *p* < 0.001; ** *p* < 0.01; * *p* < 0.05.

**Table 5:** Results of the structural equation model for problematic alcohol use.

Variable	Problematic Alcohol Use (t2) Beta Coefficient (SE)	Open-Mindedness (t2) Beta Coefficient (SE)	Conscientiousness (t2) Beta Coefficient (SE)	Extraversion (t2) Beta Coefficient (SE)	Agreeableness (t2) Beta Coefficient (SE)	Negative Emotionality (t2) Beta Coefficient (SE)	Problematic Alcohol Use (t3) Beta Coefficient (SE)	Open-Mindedness (t3) Beta Coefficient (SE)	Conscientiousness (t3) Beta Coefficient (SE)	Extraversion (t3) Beta Coefficient (SE)	Agreeableness (t3) Beta Coefficient (SE)	Negative Emotionality (t3) Beta Coefficient (SE)
Problematic Alcohol Use (t1)	0.45 *** (0.04)	0.03 (0.04)	0.01 (0.04)	0.03 (0.04)	−0.09 * (0.04)	0.06 (0.04)	—	—	—	—	—	—
Open-Mindedness (t1)	−0.06 (0.05)	0.57 *** (0.04)	0.13 ** (0.05)	0.14 ** (0.05)	0.32 *** (0.05)	−0.13 ** (0.05)	—	—	—	—	—	—
Conscientiousness (t1)	−0.07 (0.07)	−0.04 (0.06)	0.44 *** (0.06)	0.02 (0.07)	−0.21 ** (0.07)	0.05 (0.07)	—	—	—	—	—	—
Extraversion (t1)	−0.00 (0.05)	0.08 (0.04)	0.00 (0.05)	0.35 *** (0.05)	0.01 (0.05)	0.07 (0.05)	—	—	—	—	—	—
Agreeableness (t1)	0.00 (0.06)	0.12 * (0.05)	−0.08 (0.06)	−0.03 (0.06)	0.35 *** (0.06)	0.09 (0.06)	—	—	—	—	—	—
Negative Emotionality (t1)	0.03 (0.06)	0.08 (0.05)	−0.05 (0.06)	−0.01 (0.06)	−0.07 (0.06)	0.50 *** (0.06)	—	—	—	—	—	—
Problematic Alcohol Use (t2)	—	—	—	—	—	—	0.35 *** (0.04)	0.03 (0.04)	−0.05 (0.04)	−0.00 (0.04)	−0.05 (0.04)	0.05 (0.04)
Open-Mindedness (t2)	—	—	—	—	—	—	−0.08 (0.06)	0.43 *** (0.05)	−0.05 (0.05)	0.12 * (0.05)	−0.02 (0.05)	0.11 * (0.05)
Conscientiousness (t2)	—	—	—	—	—	—	0.01 (0.06)	0.19 ** (0.06)	0.56 *** (0.05)	0.09 (0.06)	0.19 ** (0.06)	−0.11 (0.06)
Extraversion (t2)	—	—	—	—	—	—	0.07 (0.05)	0.06 (0.05)	0.03 (0.05)	0.41 *** (0.05)	−0.04 (0.05)	−0.08 (0.05)
Agreeableness (t2)	—	—	—	—	—	—	−0.19 ** (0.06)	−0.06 (0.06)	−0.01 (0.06)	0.03 (0.06)	0.40 *** (0.05)	−0.02 (0.06)
Negative Emotionality (t3)	—	—	—	—	—	—	0.01 (0.06)	0.09 (0.06)	−0.01 (0.06)	0.04 (0.06)	−0.02 (0.06)	0.44 *** (0.06)
*R* ^2^	0.23	0.36	0.26	0.19	0.20	0.20	0.19	0.29	0.32	0.27	0.29	0.29

Note. Model fit: RMSEA = 0.08;, SRMR = 0.04; CFI = 0.96; TLI = 0.85. t1 = time point 1; t2 = time point 2; t3 = time point 3. SE = standard error. *** *p* < 0.001; ** *p* < 0.01; * *p* < 0.05.

**Table 6:** Results of the structural equation model for the three problematic behavioral patterns.

Variable	Internet Gaming Disorder (t2) Beta Coefficient (SE)	Problematic Social Media Use (t2) Beta Coefficient (SE)	Problematic Alcohol Use (t2) Beta Coefficient (SE)	Internet Gaming Disorder (t3) Beta Coefficient (SE)	Problematic Social Media Use (t3) Beta Coefficient (SE)	Problematic Alcohol Use (t3) Beta Coefficient (SE)
Internet gaming disorder (t1)	0.51 *** (0.04)	0.20 *** (0.04)	0.11 * (0.05)	—	—	—
Problematic social media use (t1)	0.12 ** (0.04)	0.40 *** (0.04)	0.00 (0.05)	—	—	—
Problematic alcohol use (t1)	−0.02 (0.04)	−0.03 (0.04)	0.45 *** (0.04)	—	—	—
Internet gaming disorder (t2)	—	—	—	0.48 *** (0.05)	0.17 ** (0.06)	0.17 ** (0.06)
Problematic social media use (t2)	—	—	—	0.01 (0.05)	0.35 *** (0.05)	−0.12 * (0.06)
Problematic alcohol use (t2)	—	—	—	0.04 (0.04)	−0.02 (0.05)	0.36 *** (0.04)
*R* ^2^	0.32	0.27	0.23	0.25	0.23	0.16

Note. Model fit: RMSEA = 0.13; SRMR = 0.05; CFI = 0.93; TLI = 0.74. t1 = time point 1; t2 = time point 2; t3 = time point 3. SE = standard error. *** *p* < 0.001; ** *p* < 0.01; * *p* < 0.05.

## Data Availability

The data presented in this study are available upon request from the corresponding author in accordance with the agreements with the funder.
